# Treatment of Yellow Fever

**Published:** 1900-03

**Authors:** 


For Texas Medical Journal.

### Treatment of Yellow Fever.

The treatment in the majority of cases was as follows: If the patient's condition was such as to permit it, I usually gave him calomel, combined with bicarbonate of soda (three doses), a hot mustard foot bath, and a warm sponge bath from head to foot, frequently alcohol with the warm water; also an irrigation of the bowels with warm water, after which the patient was covered up in bed, but not too heavily. Sometimes a warm lemonade was given to promote perspiration. If the temperature is high, half hour after giving the first dose of calomel I gave the patient about ten grains of phenacetine, combined with citrate of caffein, to be repeated according to the necessities of the case.

Phenacetine is a coal tar product, produced by the action of glacial acetic acid on paraphenetidin, a body obtained from phenol. Experiments go to prove that the fall of temperature produced by phenacetine is due to a lessened production of animal heat caused by the direct influence of the drug upon the thermo-genetic centres, not necessarily accompanied by any distinct alteration of the circulation. By stimulating the heart, when full therapeutical doses are given, and the vaso-motor system, the arterial pressure is increased, and the capillary stagnation which is so pronounced in yellow fever is partially overcome. Owing to its analgesic properties phenacetine is a very useful remedy in relieving the intense frontal headache. Its antipyretic effect, or rather action, is more gradual, more prolonged, and less apt to be attended with disagreeable symptoms than any drug of its class. It does not irritate the stomach and is the safest and most efficient of the coal tar derivatives. Lepine and Guttman place it at the head of the list. By combining phenacetine with caffein citrate phenacetine is placed in the citric acid group, rendering it, if possible, still less poisonous. Apolysan replaces one atom of hydrogen, transforming the drug into a cit. phenatidin.

The combination is made because caffein stimulates the brain, gives an increase to the urinary excretion, and to the force of the pulse, and elevates the arterial pressure. It is a cardiac stimulant; and, in a measure, prevents or guards against heart failure. It takes the place of digitalis, which cannot be used in yellow fever. It never disagrees with the stomach and acts promptly. By reason of its action upon the kidneys, it, in a great measure, guards against suppression of urine.

There is no danger of poisoning by carbolic acid, however large the dose of phenacetine may be, as it contains no phenal, and whatever depressing influence the drug may have, is more than counterbalanced by the stimulating properties of the caffein citrate.

I have always caused the patients to be sponged off twice daily with hot water, or warm water and alcohol, and the bowels to be irrigated morning and night with warm water; this relieved the pain and uneasy feeling in the bowels, permitting the patient to rest more easily. Should symptoms of uræmic poisoning set in, or the urine become scant, I order a hot bath at once, as hot as the patient will bear, immersing him up to his neck in the water (this year I used half sea and half fresh water), allowing him to remain in the bath for about fifteen minutes, after which he is taken out, wrapped in dry blankets and one-half to a full teacup of green coffee tea is given with one ounce of gin.

This has the effect of producing a profuse perspiration, and in many cases a copious flow of urine was obtained, even after the action of the kidneys had been suppressed. The green coffee tea was suggested to me by A. A. Surgeon G. A. McHenry, who states that the remedy is extensively used on the Gulf coast, being a favorite of Surgeon Murray of the Marine Service.

As to drinks, some stomachs bear cold drinks well, in other cases warm drinks are more suitable. The patient calls for water at frequent intervals, this, however, should be given with caution, as there is certain danger of overcharging the stomach, and producing vomiting, which always causes the physician anxiety. A preparation of cream of tartar water, with or without lime juice, is generally very palatable to the patient, and has the advantage of not only quenching the thirst, but it also keeps the bowels open, and stimulates the action of the kidneys.

As to nourishment, if the patient can possibly hold out, I give none at all, and if obliged to do so, rice water has always been my favorite. I cannot endorse the custom of the native (Cuban) physicians of giving milk, with or without lime water or Apollinaris, the stomach cannot digest it, and does more harm than good, it is very apt to curdle and start the patient to vomiting.

Black vomit I have always controlled, when at all, better with the subnitrate of bismuth than any other remedy at my command. The great difficulty being that the doses are generally not large enough. In administering the drug in black vomit, or when it is threatened, not less than three drachms should be given at one dose, and repeated, if necessary, according to the judgment of the physician.

As to alcohol during the fever it should be given with great caution. Occasionally we may venture on cracked ice champagne in very moderate doses, when deemed absolutely necessary to keep up the patient's strength. After the fever has left the patient, in my opinion, nothing tends to help him regain his strength so rapidly as alcoholic stimulants judiciously employed. The fever is a very exhausting one, and the patient's vitality much impaired. Though the distilled liquors are not in themselves stimulants, yet they may be classed as negative articles of food, and, while adding nothing to the patient's strength, they prevent waste, and preserve the strength of the patient, enabling him to recuperate more rapidly. During the stage of convalescence, the bitter tonics, a mild preparation of iron with a good light wine, are of much benefit.

